# Coral distribution and bleaching vulnerability areas in Southwestern Atlantic under ocean warming

**DOI:** 10.1038/s41598-021-92202-2

**Published:** 2021-06-25

**Authors:** Jessica Bleuel, Maria Grazia Pennino, Guilherme O. Longo

**Affiliations:** 1 Laboratório de Ecologia Marinha, Departamento de Oceanografia e Limnologia, Universidade Federal do Rio Grande do Norte, Av. Via Costeira/Senador Dinarte Mariz s/n, Natal, RN 59014-002 Brasil; 2 Programa de Pós-Graduação Em Ecologia, Universidade Federal do Rio grande do Norte, Lagoa Nova, Natal, RN 59072-970 Brasil; 3 Instituto de Español de Oceanografía, Subida Radio Faro, 50, 36390 Vigo, Spain

**Keywords:** Coral reefs, Climate-change ecology, Ecological modelling, Conservation biology

## Abstract

Global climate change is a major threat to reefs by increasing the frequency and severity of coral bleaching events over time, reducing coral cover and diversity. Ocean warming may cause shifts in coral communities by increasing temperatures above coral’s upper thermal limits in tropical regions, and by making extratropical regions (marginal reefs) more suitable and potential refugia. We used Bayesian models to project coral occurrence, cover and bleaching probabilities in Southwestern Atlantic and predicted how these probabilities will change under a high-emission scenario (RCP8.5). By overlapping these projections, we categorized areas that combine high probabilities of coral occurrence, cover and bleaching as vulnerability-hotspots. Current coral occurrence and cover probabilities were higher in the tropics (1°S–20°S) but both will decrease and shift to new suitable extratropical reefs (20°S–27°S; tropicalization) with ocean warming. Over 90% of the area present low and mild vulnerability, while the vulnerability-hotspots represent ~ 3% under current and future scenarios, but include the most biodiverse reef complex in South Atlantic (13°S–18°S; Abrolhos Bank). As bleaching probabilities increase with warming, the least vulnerable areas that could act as potential refugia are predicted to reduce by 50%. Predicting potential refugia and highly vulnerable areas can inform conservation actions to face climate change.

## Introduction

Coral reefs are threatened by increasing ocean temperatures and extreme heating events related to human-induced global climate change^1–3^. The most notable response of corals to warming is to expel the endosymbiotic photosynthetic dinoflagellates (family Symbiodiniaceae^4^) from their tissue, losing their pigmentation and main source of nutrition, a phenomenon known as coral bleaching^5^. Globally, coral reefs have experienced four pan-tropical massive bleaching events in the last 23 years (1997–1998, 2010, 2015–2017 and 2019–2020), which are becoming more frequent and severe over time^6^. Severe bleaching events are predicted to start occurring annually before 2055^7^, and bleached corals to get more susceptible to diseases and mortality^8, 9^. Repeated bleaching events can reduce the thermal tolerance^10^ and recovery capacity of corals^11, 12^, which can lead to declines in coral cover and biodiversity loss^13, 14^.

Reef biodiversity is threatened by warming as oceans are predicted to warm between 1 and 5 °C on average by the end of this century (IPCC, 2018). Warming affects the physiology and phenology of marine organisms, including their dispersal potential, causing range shifts through expansion or contraction of species distribution^15, 16^. The physiological limit of most zooxanthellate scleractinian corals is between ~ 18 and ~ 28 °C^17^. As oceans warm, some regions might exceed this upper physiological limit (˃28 °C) and become less suitable for corals^18^, while other regions might increase their minimum temperature and become more suitable for corals to thrive (˃18 °C; tropicalization *sensu*^19^). Tropical reefs could become too warm, leading to local declines, while extratropical reefs may become more suitable, as temperatures in these areas will approach the current tropical average^18^. The survival of coral species in a warmer future will likely depend on their ability to disperse to thermal refuges, where temperatures remain tolerable or become more suitable (e.g. extratropical, deeper reefs, marginal reefs^20^).

Marginal reefs are located outside the shallow, clear and warm water areas closer to the equator, and occur under suboptimal conditions, such as high turbidity, cold and upwelling waters, often not tolerated by most corals^20, 21^. Southwestern Atlantic reefs, for instance, can be considered marginal because they are highly influenced by river discharges resulting in high sedimentation and nutrient concentration, and low light conditions, comprising an impoverished but highly endemic coral fauna and a potential refugia for corals^20, 22^. Extratropical reefs can also be considered marginal and potentially act as refugia since it experiences less intense thermal stress events^23^ and annual severe coral bleaching events are expected to start years later in comparison to tropical reefs^7^. Corals in mesophotic reefs can also experience less bleaching due to lower light availability and milder temperatures^24, 25^. Identifying potential refugia, such as marginal and mesophotic reefs, is critical to understand coral resilience under future scenarios.

Although mesophotic (~ 40 m-100 m) offshore reefs could theoretically be important refugia, there is still a large debate on such assumption because coral communities are often different from those in shallow reefs (< 30 m deep^26^), which could limit the vertical connectivity between these communities^27^. Such disparity could be context-dependent as species overlap between coral communities in the upper mesophotic zones (~ 30 m-60 m deep) and shallower zones (< 30 m deep^25^), and vertical connectivity depend on the coral species and local environmental conditions^28, 29^. Coral species in Brazilian reefs, for instance, are more generalist in their coral-endosymbiont relationships and have wider depth ranges^20^, reducing the disparities between the shallow and the upper mesophotic zones mainly for major reef builders (e.g.* Montastraea cavernosa*, *Siderastraea stellata*^30^), which could enhance the role of some mesophotic reefs as refugia.

Corals could also acclimatize to climate change through epigenetic processes, in which the phenotype of a new generation is influenced by the environmental conditions that previously influenced past generations (transgenerational plasticity^31^). In addition, corals might adapt to climate change through their associated symbionts and microbiota (i.e. holobiont), enhancing the holobiont adaptive capacity^31,32^. Coral acclimatization to warmer waters through generations is more likely to occur where local human impacts, such as pollution, overfishing, and nutrient enrichment, are minimized^33,34^. Thus, identifying the most vulnerable areas to coral bleaching could inform actions to minimize local impacts and reduce threats from global stressors^35, 36^.

Several studies have used climate models to predict coral bleaching, using different approaches across time and scale (reviewed by^37^), including vulnerability to thermal stress and local anthropogenic impacts^38^, and a combination of frequency and severity of coral bleaching, resilience and human impact^39^. We used a novel framework to assess reef vulnerability to coral bleaching, by combining spatial projections of coral occurrence, relative abundance and bleaching percentage across a large spatial scale to identify bleaching vulnerability-hotspots under warming scenarios (i.e. areas that combine high probabilities of coral occurrence, cover and bleaching).

We modeled coral occurrence, cover and bleaching probabilities across 28° of latitude in the Brazilian coast that harbors the largest and richest marginal reefs in Southwestern Atlantic^20^, and predicted how these probabilities are likely to change by 2040–2050 and 2090–2100, under the high-emission scenario *Representative Concentration Pathway 8.5* (RCP8.5) forecasted by the Intergovernmental Panel on Climate Change (IPCC). We identified vulnerability patterns under current and future scenarios, assuming that higher probabilities of coral occurrence, cover and bleaching combined indicate a vulnerability-hotspot (highly vulnerable area to coral bleaching), and areas with low coral occurrence, cover and bleaching probabilities indicate least vulnerable areas. We hypothesized that: (i) high coral occurrence and cover in the tropics will decline with future warming because temperature conditions are likely to exceed corals’ thermal limits; (ii) corals will shift or expand their distributions toward extratropical and deeper offshore reefs, as temperatures on these reefs under future warming will remain within the optimum temperatures of zooxanthellate corals; (iii) higher vulnerability areas will be concentrated within the tropical region in areas with high coral cover, where corals are closer to their upper thermal threshold, enhancing coral bleaching probability. This modelling approach to identify current and future vulnerability-hotspots can be a powerful tool to guide conservation actions.

## Results

**Figure 1:**
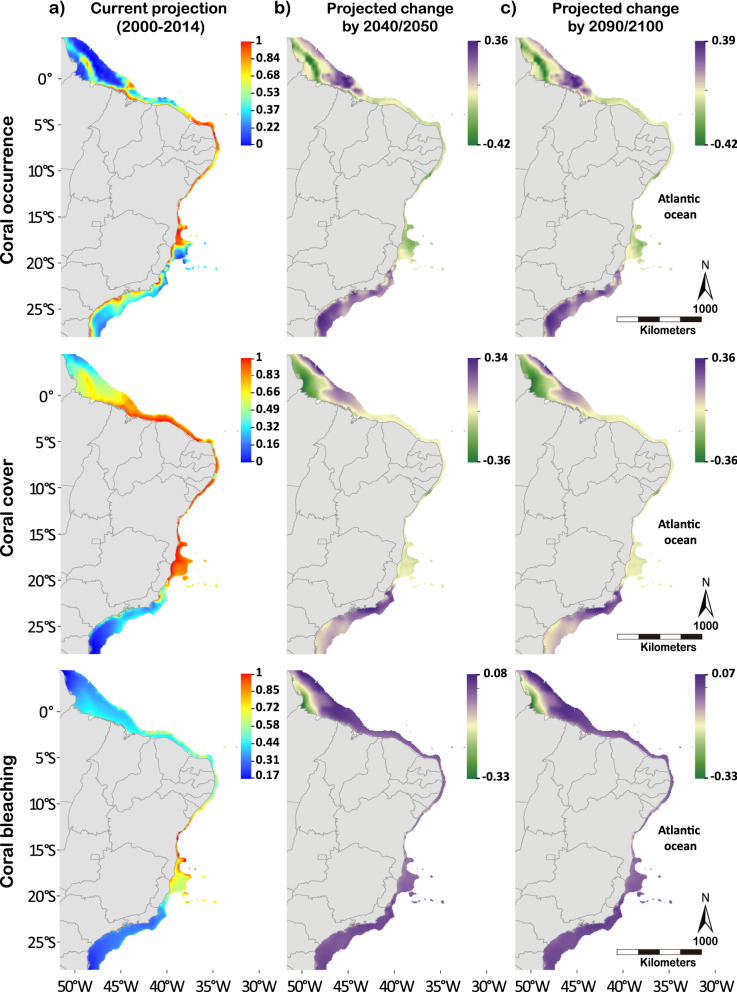
Probability maps for current projections and delta maps of projected changes between current and future scenarios of coral occurrence, coral cover and coral bleaching along the Southwestern Atlantic coastline (1°N–27°S latitude). (**a**) The probability maps for coral occurrence, cover, and bleaching for the current scenario (2000–2014) and (**b**,**c**) delta maps between current and the future scenarios (2040–2050 minus 2000–2014, left; and 2090–2100 minus 2000–2014, right) under a “business as usual” warming rate (RCP 8.5). The blue-red and green-purple scale bars represent absolute probability values and delta probability values, respectively. Maps created in ArcMap version 10.2 (https://desktop.arcgis.com/en/arcmap/).

**Figure 2:**
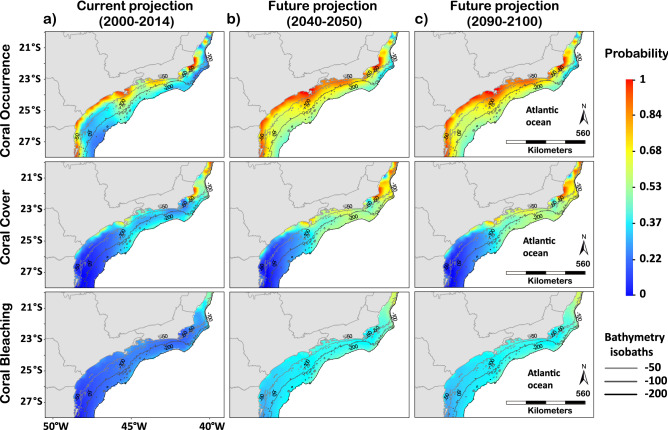
Current and future projections of coral occurrence, coral cover and coral bleaching in the transitioning zone from tropical to subtropical areas in the Brazilian province (20°S–27°S). (**a**) Current and (**b**,**c**) future probability projections of the transitioning zone from the tropical to the subtropical region with bathymetry isobaths represented by the different contours, from 50 to 200 m depth, which are specified in the legend (gln_batimetria_cprm_30 shapefiles, from (https://geoservicos.inde.gov.br/geoserver/web/). The blue-red scale bar represents absolute probability values. Maps created in ArcMap version 10.2 (https://desktop.arcgis.com/en/arcmap/).

The current projections indicate a high probability of coral occurrence, cover, and bleaching within the tropics (lower limit at 20°S; Fig. 1; Fig. S1). These probabilities will expand towards the subtropical region (beyond 20°S) and offshore reefs (deeper) in future scenarios under a “business as usual” warming rate (high-emission scenario RCP 8.5; Fig. 2). Coral occurrence showed the greatest changes of probability from current to future scenarios in comparison to coral cover and bleaching probabilities (Fig. 1b,c; Table S1). Coral occurrence and mean coral cover probabilities increased poleward with warming (beyond 20°S), however only coral occurrence decreased between 1°S and 20°S of latitude, while there was almost no change for coral cover (Fig. 1). Projections of coral bleaching probability followed the same southward and offshore shifts but with a subtle increase across the entire modeled area (Fig. 1b,c). Combined, these future projections revealed new suitable areas for corals in subtropical Brazil, particularly within latitudes of 20°S and 27°S (Fig. 2).

**Figure 3:**
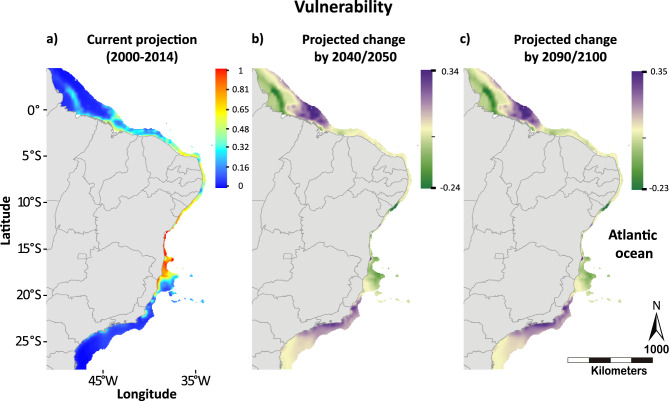
Probability map of vulnerability for the current scenario and delta maps of projected changes between current and future scenarios. (**a**) Vulnerability areas based on the overlap of coral occurrence, coral cover and coral bleaching probabilities of the Brazilian coast for the current (2000–2014) scenario and (**b**,**c**) delta maps between current and the future scenarios (2040–2050 minus 2000–2014, left; and 2090–2100 minus 2000–2014, right) under a “business as usual” warming rate (RCP 8.5). The blue-red and green-purple scale bars represent absolute probability values and delta probability values, respectively. Maps created in ArcMap version 10.2 (https://desktop.arcgis.com/en/arcmap/).

The vulnerability-hotspots represent ~ 3% of the area and are concentrated between latitudes 13°S and 20°S, which comprises several reef habitats within the most diverse reef system in South Atlantic, the Abrolhos Bank (17°S; (Fig. 3; Fig. S2). Areas considered as vulnerability-hotspots remain within the same latitudes in future scenarios with almost no change in intensity, while areas in the Northern coast (1°S–4°N) and in Southern Brazil (20°S–27°S) tend to increase in vulnerability with warming (Fig. 3; Fig. S2). Based on the quartiles of the vulnerability distribution (Fig. S2), more than 80% of the area in the current projection are under intermediate vulnerability to coral bleaching (> 0.22 and < 0.77), 12.6% are under lower vulnerability (< 0.21), and only 3.13% have higher values (> 0.78; Fig. 3) indicating a vulnerability-hotspot. However, given the future warming, lowest vulnerability areas could experience a 50% reduction by the end of the century as they increase in vulnerability (Fig. 3; Fig. S2). The region between latitudes 20°S and 27°S, in turn, had the lowest vulnerability in all scenarios (refugia), even though this region is predicted to experience an increase in vulnerability with warming.

## Discussion

By combining spatial modelling approaches, we created a novel framework to identify areas of vulnerability to coral bleaching and to predict changes in these areas in future warming scenarios, accounting for changes in species distribution, abundance and bleaching probabilities. The steps to identify vulnerability-hotspots include projecting coral occurrence, cover and bleaching probabilities, and overlapping these three predictions. We documented an increase in coral occurrence and cover towards extratropical and deeper offshore reefs by 2040–2050 and 2090–2100 in Southwestern Atlantic, indicating that these areas may act as refugia under warming scenarios. However, the simultaneous increase in the vulnerability to coral bleaching also detected in these areas by 2040–2050 and 2090–2100 associated to potential limitations in vertical connectivity between shallow and deeper offshore reefs^27, 30^, could challenge the effectiveness of these refugia. Vulnerability-hotspots were concentrated around the largest and richest coral reef area in South Atlantic, the Abrolhos Bank (13–18°S^40, 41^), that comprises a network of marine protected areas^42, 43^ that may mitigate local stressors and enhance the ability of corals to cope with rising temperatures^34^. These modeling approaches combined can be a powerful tool to inform conservation actions, accounting for future range expansion and vulnerability to bleaching.

**Figure 4:**
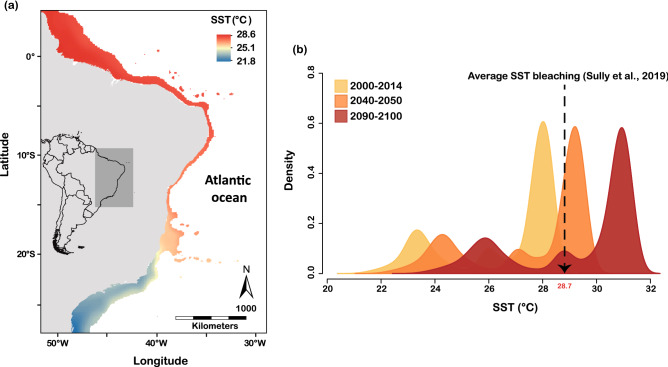
Current and future sea surface temperature (SST) of the Brazilian coast from extracted values of the Bio-ORACLE database. (**a**) Current mean annual SST at the Brazilian coast and (**b**) the density of mean SST extracted along the Brazilian coast for current (2000–2014), and future 2040–2050 and 2090–2100 projected SST under a “business as usual” warming rate (RCP 8.5). The vertical dotted line and arrow points the average threshold SST (28.7 °C) recorded during global coral bleaching events between 2007 and 2017 (Sully et al., 2019). Note that the majority of the regions of mean SST for 2040–2050 and 2090–2100 will exceed mean SST bleaching for the current decade. Density was performed using the Kernel Density Estimation. The Map was created in ArcMap version 10.2 (https://desktop.arcgis.com/en/arcmap/) and the density graph was plotted using the package “yarrr”^49^ in R software^50^.

Most coral reefs in the world are within tropical regions, however several organisms are shifting their distribution poleward in response to warming and corals are likely to do the same^44^. Poleward expansion due to temperature changes has happened with Acroporid corals in Japan^45^ and in the east coast of the USA (Florida^46^). Along the Southwestern Atlantic coastline, the highest coral cover is located within latitudes 13°S and 18°S resulting from a combination of suitable tropical conditions and a wider continental shelf in comparison to other areas along the coast^40, 47^. Tropical regions tend to be more suitable for zooxanthellate scleractinian corals because temperatures fit their physiological limits but may exceed these limits as ocean warms (Fig. 4), while temperature in subtropical reefs can be suboptimal or even below tolerable levels to corals^48^. The increase in temperature of subtropical regions under future climate change scenarios will create conditions that are more similar to what is currently experienced in tropical areas (i.e. tropicalization), enabling range expansion of tropical species into these subtropical areas^19^.

As subtropical areas become warmer, they could serve as coral refugia, with more suitable temperature conditions. Corals in subtropical Brazilian reefs experienced low mortality in one of the most severe bleaching events in South Atlantic^51^, indicating their potential as refugia if habitat suitability increases. Other modeling studies have predicted poleward expansion of corals, including the Brazilian endemic species *Mussismilia harttii* currently restricted to tropical reefs but likely to expand its distribution towards subtropical reefs as ocean warms^52^. Similarly, two *Acropora* species in the Caribbean have expanded more than 50 km poleward in the last decade^46^. The poleward expansion of corals occurs during the larval periods and depends on ocean currents and habitat suitability^53^. Recent evidences point to a decline in coral recruitment in tropical regions and an increase in extratropical regions^54^, demonstrating the potential for distribution shift. Contrary to terrestrial species, range expansions are more likely and occur faster in marine species, which tend to have longer dispersal periods in the water column and higher sensitivity to temperature change^16^. Therefore, new suitable habitats for corals could function as refugia to climate change along the Southwestern Atlantic, as observed in the East and West coast of Australia for the coral *Porites lobata*^55^ and Acroporid corals in the Caribbean^46^ and Japan^45^.

Our future projections also indicated mesophotic offshore reefs as critical areas for future coral occurrence and abundance, which suggests their potential as refugia^56^. However, there is no consensus on the role of mesophotic reefs as refugia^26, 30, 57^ but see^56^). The upper mesophotic (~ 30 m-60 m) and shallower zones (< 30 m) might overlap their communities to some extent, being suitable habitats as refugia^25^. However, deeper mesophotic zones (~ 60 m-100 m) and shallow communities (< 30 m) can differ to the extent of almost no species overlap^26^, suggesting that vertical connectivity between these communities may not occur even under future climate scenarios^27^. Mesophotic offshore reefs, such as those indicated in our model, might be less exposed to local threats that affect shallow nearshore reefs (e.g. sedimentation and pollution), and experience less severe temperature changes under future scenarios, providing a reproductive shelter to warming. However, the success of recruits on these hypothetical refugia is yet to be determined. Connectivity studies in the Caribbean have shown that shallower zones distant apart from each other may be more connected than shallower and deeper reef zones in the same area, demonstrating the difficulty of vertical connectivity for some coral species in particular regions (*Montastraea cavernosa* and *Porites astreoides*^28, 29^). Both studies still indicate significant vertical connectivity in some localities, suggesting that connectivity will depend on the species and local environmental conditions (but see^27^). Marginal reefs along the Brazilian coast are inhabited by depth generalist corals and an impoverished coral fauna^20^, where the same coral species often occur in both shallow and deeper sites (e.g.* M. cavernosa*). These marginal reefs with depth generalist corals may favor the deep reef refugia hypothesis in response to warming ocean temperatures (but see^30^). This hypothesis could also be interpreted as a “species refugia”, in which not necessarily deep reef corals are reproductive sources for shallow reef corals, but corals of deeper sites will be more likely to survive the increasing temperatures and global climate change impacts, such as bleaching events. Whether poleward latitudes (extratropical or subtropical zones) and mesophotic reefs will function as refugia will also depend on future bleaching probabilities, synergic anthropogenic impacts, and species resistance and resilience to these multiple stressors.

Alternatively, corals’ symbionts and associated microbiota (i.e. holobiont) can respond to climate change by mechanisms such as acclimatization and adaptation^16^. Rapid microbiome acclimatization or adaption due to high rates of microbial turnover and acquisition of new genes via such mechanisms as horizontal gene transfer might provide opportunities for the holobiont to survive rapid global change^31,32﻿^. If this happens at a sufficient pace to respond to climate change, corals and their symbionts may have the capacity to acclimatize their thermal threshold to live and survive in higher temperatures^58^. This adaptive response might be already happening, as corals increased their bleaching threshold temperature by almost a half-degree Celsius in a decade^48^. Corals might be able to survive and acclimatize in regions that are already under high bleaching probabilities and that will experience increasing temperatures and higher vulnerability in the future. However, coral adaptation to warmer waters has higher chances to occur in more optimistic greenhouse gas concentration (RCPs 2.6 and 6.0). Therefore, for corals to have a greater chance to survive, it is imperative that greenhouse gas emissions are reduced^59^ and that local impacts are minimized^60^.

A small portion of Southwestern Atlantic reefs (~ 3%) was identified as a bleaching vulnerability-hotspot, indicating its potential as a refugia^20^. However, this refugia will also depend on the ability of corals to colonize and thrive in new suitable areas, and on the maintenance of the low post-bleaching mortality documented in this region^20^, because the main vulnerability-hotspot in Southwestern Atlantic includes the largest and richest reef systems in this area, the Abrolhos bank, that comprise high coral cover and endemism levels (~ 30%), and diverse reef morphologies^40, 41, 43^. The Abrolhos region comprises several Marine Protected Areas (MPA), including no-take zones protected from fishing and other exploitation activities, multiple-use areas where extractive activities are regulated, and non-protected areas with open access^42^. Still, the inclusion of other adjacent marine habitats could enhance connectivity among MPAs and increase the network’s efficacy^42, 43^. For example, recently discovered reefs with significant coral cover adjacent to the Abrolhos Bank^61^ are within the vulnerability-hotspot, but are not under any kind of protection or regulatory status. In general, corals in the Abrolhos Bank have shown high recovery capacity after bleaching events (except for hydrocorals^62,63^), likely resulting from a combination of minimized local impacts and the high turbidity in the region, which can reduce intense light incidence in the water, helping corals to recover from bleaching^20, 64^. While both protected and unprotected areas will be equally exposed to heat stress and global change stressors^65^, the establishment of marine protected areas may enhance reef resilience by mitigating local impacts and allowing for corridors of connectivity among reef organisms^36, 66,34^.

The framework using Bayesian Hierarchical Spatial modeling presented in this study is a powerful predictive tool that employs known biotic and abiotic relationships (e.g. coral distributions, physiological tolerances, and temperature) to interpolate to areas that are currently under-sampled and to future reef conditions by using IPCC projections. With this approach, we were able to project coral occurrence, cover, and bleaching probabilities across 28° degrees of latitude along the Brazilian coastline under current (2000–2014) and future environmental conditions under a high-emission scenario (2040–2050 and 2090–2100, RCP8.5). However, there are some caveats that need to be addressed. Our coral occurrence and mean cover models do not account for interactions with other organisms (biological resistance) that could influence range shifts and coral abundance. We were only able to use future projections of temperature and salinity in our modelling, excluding other important variables (e.g. pH, NPP) whose projections were not available in the BIO-ORACLE and MARSPEC databases. The pH in Brazilian reefs, for instance, currently varies between 8.05 and 8.08. Under RCP 8.5 in 2080–2099 this pH could decrease between 0.30 and 0.32, varying between 7.75–7.73 to 7.78–7.76 (minimum–maximum, respectively^67^). This decrease in pH could have effects on calcification and coral growth, which would affect coral cover over time. Still, the use of pH and aragonite saturation projections to infer calcification in the future has also being criticized as it depends on large assumptions, particularly in shallow coastal ecosystems^68^. We understand that not including pH and other variables also comprise large assumptions but it makes our projections more conservative and we still observe an increase in coral bleaching probabilities in the future. Therefore, the addition of other parameters such as pH and NPP in future models is likely to generate an even worse scenario of coral vulnerability. Our input data on coral bleaching is somewhat restricted (Fig. S3), which could be related to the paucity of monitoring effort but most likely to the fact that Southwestern Atlantic reefs have suffered 50–60% fewer bleaching events with coral mortality in comparison to the Indo-Pacific and Caribbean reefs^20^. Therefore, we believe this association between relatively few coral bleaching data and the BHSM tool that deals well with data-poor situation is useful to generate insights. Also, our models do not account for differences in species thermal tolerance, acclimatization or adaptation processes, which could increase coral species resistance to bleaching events and influence bleaching probabilities^48^. Such information is still scarce particularly for Southwestern Atlantic corals but will be of great value if future modeling approaches are able to account for it^69^.

We predicted that tropical coral species might expand their ranges toward extratropical reefs and into deeper waters that may serve as refugia to climate change in South Western Atlantic. However, as coral bleaching and vulnerability probabilities are expected to increase in these areas with warming, the role of subtropical and deeper offshore reefs as refugia might be challenged. Our framework to identify vulnerability areas to coral bleaching is a useful tool to inform conservation actions to minimize local impacts on vulnerability-hotspots and future potential refugia, and to enhance connectivity among them. These informed actions may enhance our ability to help corals to cope with global climate change.

## Methods

### Study area

The Brazilian province holds the largest marginal reefs in the Southwestern Atlantic, subject to river outflows, resulting in high turbidity and nutrients in the water column^20, 22^. Brazilian reefs occur from 1°N to 27°S of latitude, comprising a wide variation in reef cover^47^ and mean annual sea surface temperatures, which varies between ~ 21 and ~ 28 °C (Fig. 4). The heterogeneity in reef structure and abiotic conditions in Southwestern Atlantic reefs provide an opportunity to model reef vulnerability to bleaching and predict vulnerability-hotspots under warming scenarios.

### Coral dataset

We assembled a dataset on coral occurrence, cover and bleaching percentage of 23 shallow water zooxanthellate species (19 scleractinian corals and 4 hydrocorals) by searching information published in journal articles, reports, dissertations, and thesis searched on Web of Science, Google Scholar, CAPES portal (Brazilian Ministry of Education), and universities repositories. We used keywords related to coral occurrence, cover, bleaching, and monitoring, such as: “Brazilian coral fauna”, “coral bleaching”, “coral monitoring” and coral species names (see Table S2). This search resulted in 37 publications to which we added other 14 that did not appear on the search but were cited in at least one of the 37. After processing the 51 publications, we were able to obtain data from 33 covering 118 sites within 45 localities across 28° of latitude along the Brazilian coast, spanning from 1993 to 2017 (see details in Table S3 and Fig. S3). For each site we had a list of presence and absence of coral species and modeled them combined as an assemblage to project occurrence probabilities for current and future ocean conditions. We used relative abundance and relative percentage of bleached corals of each species per site, but modelled species combined as an assemblage to project cover and bleaching probabilities, respectively. When coral cover data per species was presented as fraction of total coral cover without accounting for other organisms in benthic community, we recalculated it considering the relative abundance of corals in the study area.

### Environmental data

The current projections of coral occurrence, cover, and bleaching probabilities were based on the annual mean of six oceanographic predictors from 2000 to 2014 obtained from the Bio-ORACLE database^70, 71^ (see details in Supplementary information): sea surface temperature (SST), sea surface salinity (SSS), diffused attenuation coefficient (KD), pH, dissolved oxygen (O_2_), net primary productivity (PP). Two topographic variables were obtained from MARSPEC^72^: bathymetry and rugosity (Table S4). Rugosity was derived from bathymetry maps applying the “terrain” function in the "raster”^73^ package in R software^50^, which measures the seabed slope^74^. All variables were standardized and transformed to the same spatial resolution based on the lowest resolution within our environmental data (0.08 × 0.08 decimal degrees) using the “raster”^73^ package in the R software^50^.

For future projections, we used annual SST and SSS means extracted from the Bio-ORACLE database under the “business as usual” warming rate (RCP8.5) for the two periods, 2040–2050 and 2090–2100. SST and SSS are expected to increase 1 °C and 1 PSU, and almost 3 °C and 1.5 PSU on average by 2050 and 2100, respectively^75^. We used the RCP8.5 scenario due to no clear indication of significant reductions in CO2 emissions that could prevent this extreme scenario to happen. Based on the RCP8.5 predictions, all other environmental variables used in the present-day models were maintained constant for future projections, as future estimates of these variables were not available at the Bio-ORACLE and MARSPEC databases.

### Modeling approach

We applied Bayesian Hierarchical Spatial Models (BHSMs) to project coral occurrence, cover and bleaching probabilities along the Brazilian coast under current (2000–2014) and future conditions (2040–2050 and 2090–2100, RCP8.5). The BHSMs approach has the advantage of interpolating the biological data to non-sampled areas due to correlations between biological and environmental data, which is a useful tool in data-poor situations such as coral bleaching events that are not necessarily reported or available for several locations (e.g. South Atlantic^20^). In addition, this was used due to expected relationships between biological and environmental data, and the influence of covariates on coral distribution, abundance, and bleaching.

Correlation among environmental data were explored using the Pearson’s correlation index, and multicollinearity was checked with the generalized variance inflation factor (GVIF) using the “covif” function^76^ in R software^50^. GVIF was corrected to the common VIF by the number of degrees of freedom of the predictor variable^76^. We selected the environmental variables that were statistically and biologically suitable for our model by removing variables step-by-step with GVIF higher than 3 and a correlation higher than 0.70^77^ (see variables used in each model in Table S5).

By establishing the relationship between biological data and environmental variables in space under present conditions, we predicted their current distribution in un-sampled sites and under climate change scenarios. The BHSMs implemented here are similar to a spatial extension of a General Linear Model (GLM), as the modeling process describes the variability in the response variable as a function of the explanatory variables, with the addition of a stochastic spatial effect, which models the residual spatial autocorrelation. In particular, the coral occurrence dataset was modeled with a Bernoulli distribution and a logarithmic link function, where the response variables Y_i_ represent the process occurrence (1 for presence; 0 for absence) at each sampled location *i*.

For the coral cover dataset, we used a two-step model, modeling the proportion of coral cover conditional to the species presence. In this model Y_i_ and Z_i_ denote, respectively, the spatial distributed species occurrence and the conditional-to-presence species cover, where *i* = 1,…, n is the spatial location. Then, we modeled the occurrence, Y_i_, using a Bernoulli distribution as for the coral occurrence. In the case of the coral cover and bleaching percentage, Z_i_, we used a Lognormal distribution. The mean of both variables was then related via the usual link functions (logit and log, respectively) to the environmental effects:

1 $${\text{Y}}_{{\text{i}}} \sim \,{\text{Bernoulli}}\,({\pi }_{{\text{i}}} )$$

2 $${\text{Z}}_{{\text{i}}} \sim \,{\text{Lognormal}}({\mu }_{{\text{i}}} ,{\sigma }_{{\text{i}}} ^{2} )$$

$$\begin{aligned} & {\text{logit}}\,({\pi }_{{\text{i}}} ) = {\alpha }^{{\left( {\text{Y}} \right)}} + {\text{X}}_{{\text{i}}} {\beta } + {\text{W}}_{{\text{i}}} ^{{({\text{Y}})}} \\ & \log \,\left( {{\mu }_{i} } \right) = {\alpha }^{{\left( {\text{Z}} \right)}} ~ + {\text{X}}_{{\text{i}}} {\beta } + {\text{W}}_{{\text{i}}} ^{{({\text{Z}})}} \\ \end{aligned}$$

where π_i_ represents the probability of occurrence at location *i* and µ_i_ and σ^2^ are the mean and variance of the conditional-to-presence coral cover. The linear predictors containing the effects to which these parameters π_i_ and µ_i_ are linked are formed with: α^(Y)^ and α^(Z)^, the terms representing the intercepts for each variable; β is the vector of regression parameters, X_i_ is the matrix of the explanatory covariates at location *i*, and the final terms W_i_^(Y)^ and W_i_^(Z)^ refer to the spatial structure of the occurrence and conditional-to-presence coral cover, respectively. For all models, Bayesian parameter estimates and predictions were obtained through the Integrated Nested Laplace Approximations (INLA^78^) approach^79^ implemented in the R software^50^.

For the spatial effects (W), INLA implements the Stochastic Partial Differential Equations (SPDE) approach^80^, that involves the approximation of a continuously indexed Gaussian Field (GF) with a Matérn covariance function by a Gaussian Markov Random Field (GMRF). A prior Gaussian distribution with a zero mean and covariance matrix was assumed for the spatial component (see^81^, for more detailed information about spatial effects). As recommended by^82^, vague zero-mean Gaussian prior distribution with a variance of 100 were assigned for all fixed-effect parameters, which are approximations of vague priors designed to have little influence on the posterior distributions.

### Model selection

The null model was used as a basal model (intercept without spatial effect), adding covariates afterward one by one. For each model we compared values of the Watanabe-Akaike information criterion (WAIC)^83^, and the average logarithmic score of the Conditional Predictive Ordinate (LCPO)^84^. The best model was selected based on the lowest values of WAIC and LCPO combined (Table S6), which confer the best-fit and the best predictive quality of the model, respectively^85^ (see Table S7 for the numerical summary of the posterior distributions of the fixed effects for the best model of the coral occurrence, coral cover, and coral bleaching). Therefore, model’s accuracy was assessed through the LCPO which is a “leave-one-out” cross-validation index to assess the predictive power of the model^86^. Through this, we obtain a value of the sum of all the failure vectors of our model going from 0 to 1 (values closer to 0 indicate better models; see Table S6). All variables selected in the models showed a probability to be different from zero varying between 63 and 99%, indicating their degree of relevance in determining the studied processes (Table S8). We acknowledge that while some variables had a minor predictive power (e.g. some of them had a probability to be different from 0 lower than 0.90) we opted to keep them in the models due to their relevant contribution to the studied process highlighted by the lowest WAIC and LCPO values.

### Model prediction

Based on the approximate Bayesian inference, we predicted coral occurrence, cover, and bleaching probabilities to non-sampled areas of interest using additional functions that linearly interpolate the results from observed locations^81^. Using the established models, we computed future predictions under RCP8.5 using SST and SSS for 2040–2050 and 2090–2100, while keeping the rest of the variables constant as no future predictions were available for them.

### Vulnerability of areas to coral bleaching: finding the hotspots

We overlapped projections of coral occurrence, cover, and bleaching probabilities as contributing conditions to bleaching vulnerability. All projections were standardized by its maximum (resulting in variation between 0 and 1) prior to the overlap to avoid disproportional weights. The overlap (multiplication) of the three projections resulted in vulnerability values varying between 0 and 1, in which the higher the value, the higher is the chance of vulnerability in probabilistic terms. Vulnerability-hotspots to coral bleaching were defined as regions where the probability value from the overlapped models (i.e. vulnerability) were in the upper quartile of the probability distribution of the current projection (≥ 0.78; Fig. S2). Therefore, regions that combined high probability of coral occurrence, cover and bleaching were considered the most vulnerable areas (vulnerability-hotspots), because bleaching events in these areas could affect more species, have a greater effect in the ecosystem due to high coral abundance, and because bleaching events would be more likely to occur (higher probabilities). Regions with low coral cover and occurrence, and high bleaching probabilities would have intermediate vulnerability, because corals potentially have a lower relative importance to the reef structure in these areas. In contrast, less vulnerable areas could have high or low coral cover and occurrence, with low bleaching probability. The Kernel Density Estimation were used to visualize and compare the distribution of our models among different scenarios, and differences were represented by the percentage of change between current and future projections^87^.

## Supplementary Information

Supplementary Information.

## Data Availability

The data that support the findings of this study are publicly available in the Zenodo platform with the following identifier (10.5281/zenodo.4918332).
