# Supplementary material for: Coral distribution and bleaching vulnerability areas in Southwestern Atlantic under ocean warming
Source: Sci Rep. 2021 Jun 25;11:12833. doi: 10.1038/s41598-021-92202-2 (PMC8233347; doi:10.1038/s41598-021-92202-2)
Supplement: Supplementary file 1 — Supplementary Information. [file 41598_2021_92202_MOESM1_ESM.docx]

**Supplementary information**

**Coral distribution and bleaching vulnerability areas in Southwestern Atlantic under ocean warming**

Jessica Bleuel ^1,2^, Maria Grazia Pennino ^3^, Guilherme O. Longo ^1,2,*^

^1^Laboratório de Ecologia Marinha, Departamento de Oceanografia e Limnologia, Universidade Federal do Rio Grande do Norte. Av. Via Costeira/Senador Dinarte Mariz s/n – 59014-002 Natal, RN, Brasil.

^2^Programa de Pós-Graduação em Ecologia, Universidade Federal do Rio Grande do Norte, Lagoa Nova, 59072–970, Natal, Rio Grande do Norte, Brasil

^3^Instituto de Español de Oceanografía, Subida Radio Faro, 50, 36390, Vigo, Spain.

This Supplementary material contains detailed information on the results and methods sections. In the methods, you will find the coral dataset table and some steps we used to build the dataset, and biological aspects of the environmental variables selected for modeling. In the results, you will find more detailed information on the projected probabilities of coral occurrence, cover, bleaching and vulnerability areas along the Brazilian coast (including supplementary figures), which can potentially guide local conservation efforts. Therefore, we describe the results on a basis of state, where conservation actions could be implemented.

**Detailed Results**

**Coral occurrence, coral cover and coral bleaching**

In the current projections of coral occurrence and cover, the regions between latitudes 2°S and 20°S (from the State of Maranhão to Espírito Santo), had the highest probabilities, except for the region between eastern Maranhão and Ceará (Fig. 1 in the main text and Fig. S1). Regarding coral bleaching, the highest probabilities were concentrated between latitudes 13°S to 18°S (State of Bahia), while the region between latitudes 20°S to 28°S (from the State of Espírito Santo to Santa Catarina) had the lowest probabilities of coral occurrence, cover and bleaching (Fig. 1 in the main text and Fig. S1).

In future projections of 2040-2050 and 2090-2100, the regions with the highest probabilities of coral occurrence, cover and bleaching remained the same as the current projections (Fig. 1 in the main text and Fig. S1). However, future probabilities of coral occurrence increased southwards between latitudes 22°S to 28°S (from the States of Rio de Janeiro to Santa Catarina; Fig. 2 in the main text) and at the northern coast of the state of Maranhão (Fig. 1 in the main text and Fig. S1). There was a slight reduction in coral occurrence probabilities in the other areas of Brazilian coast (between latitudes 4°S to 18°S). Coral cover probabilities slightly decreased in most of the Brazilian coast, from latitudes 4°S to 18°S (from the states of Rio Grande do Norte to Bahia), but increased from latitudes 22°S to 25°S (from the states of Espírito Santo to São Paulo; Fig.S1). While coral bleaching probabilities increased over the entire Brazilian coast for future projections (Fig. 1 in the main text and Fig. S1). When comparing current and future projections by subtracting the current probabilities from the future probabilities, coral occurrence and coral cover had the highest differences at the southernmost region of the Brazilian coast from latitudes 22°S to 28°S (from the states of Rio de Janeiro to Santa Catarina; Fig. 1 in the main text). This also occurred at the northernmost region of the Brazilian coast from latitudes 4°N to 1°S (from the states of Amapá to Maranhão). A considerable region between latitudes 2°S to 20°S had almost no difference for both coral occurrence and cover projections.


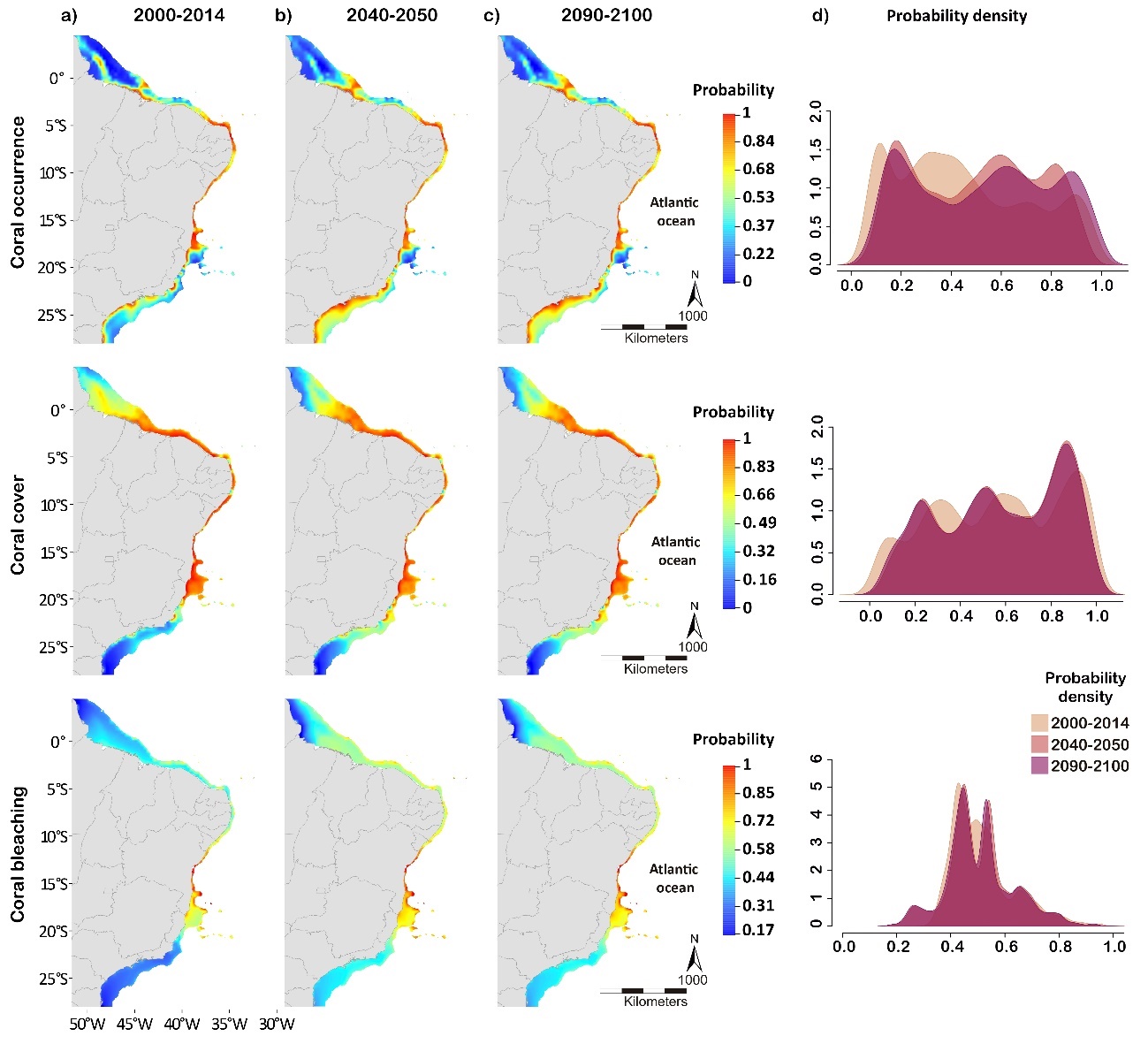


**Figure S1.** Probability maps of coral occurrence, coral cover, and coral bleaching of (a) current (2000-2014) and (b, c) future (2040-2050 and 2090-2100) scenarios under a “business as usual” warming rate (RCP 8.5) and (d) their respective overlapped density plots. The blue-red scale bar represents the absolute probability values. The density plots were performed using the Kernel Density Estimation. Maps created in ArcMap version 10.2 (https://desktop.arcgis.com/en/arcmap/) and the density graph was plotted using the package “yarrr”^1^ in R software^2^.

**Vulnerability areas**

The overlap of coral occurrence, cover and bleaching projections resulted in maps of vulnerability to coral bleaching (Fig. S2 and Fig. 3 in the main text). The coast between latitudes 13°S to 19°S (from the state of Bahia to the northern part of Espírito Santo) is indicated as a vulnerability hotspot for coral bleaching in the current and future projections (see Fig. 3, in the main text). The northern and southernmost parts of the Brazilian coast (from the state of Pará to Amapá, and Rio de Janeiro to Santa Catarina, respectively) had the lowest values for current and future vulnerability projections. The greater changes in vulnerability from current to future projections were observed at the latitude 0° (at the coast of the state of Maranhão) and from latitudes 20°S to 25°S (from Espírito Santo to São Paulo), while there were no significant changes in the other areas (Fig. S2). The regions with the highest and the lowest values of vulnerability remain the same for future projections, even with the suggestion of an increase in vulnerability southward the coast between latitudes 20°S to 25°S (from southern Espírito Santo to São Paulo; see Fig. S2).

**
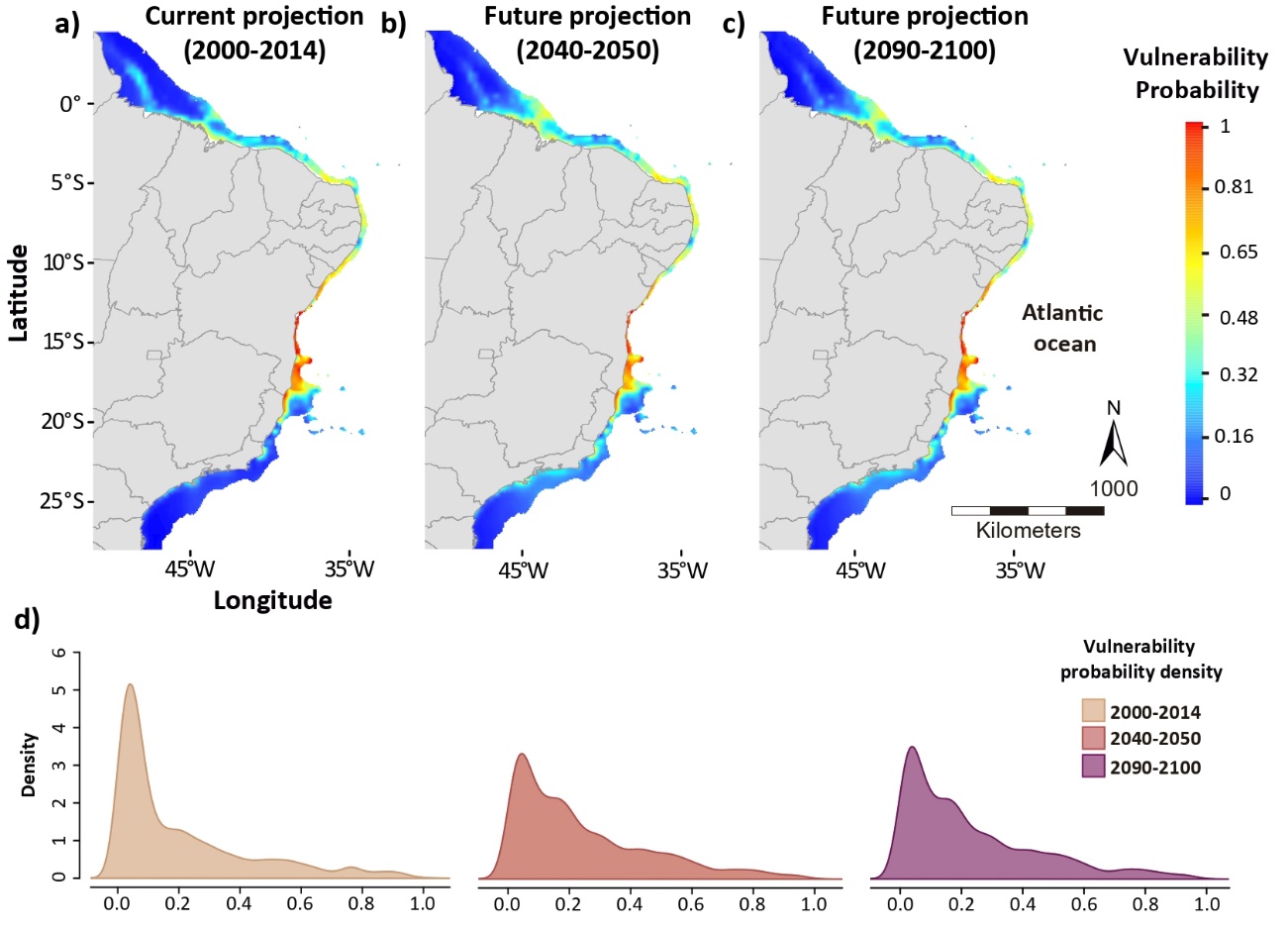
**

**Figure S2.** Vulnerability overlapped projections of (a) current (2000-2014) and (b, c) future (2040-2050 and 2090-2100) scenarios under a “business as usual” warming rate (RCP 8.5) and (d) their respective density plots. The blue-red scale bar represents the absolute probability values. The density plots were performed using the Kernel Density Estimation. Maps were created in ArcMap version 10.2 (https://desktop.arcgis.com/en/arcmap/) and the density graph was plotted using the package “yarrr”^1^ in R software^2^.

**Detailed Methods**

**Coral Dataset**

We assembled a dataset on coral occurrence, cover and bleaching percentage of 23 zooxanthellate species (19 scleractinian corals and 4 hydrocorals) based on published literature and using keywords related to coral occurrence, cover, bleaching, and monitoring, such as: “Brazilian coral fauna”, “coral bleaching”, “coral monitoring” and coral species (see Table S2). ), which resulted in 37 publications in addition to other 14 that did not appear on the search but were cited in at least one of these 37. Out of the 51 publications, we were able to obtain data from 33 covering 118 sites within 45 localities across 28° of latitude along the Brazilian coast, collected between 1993 and 2017 (see details in Table S3).


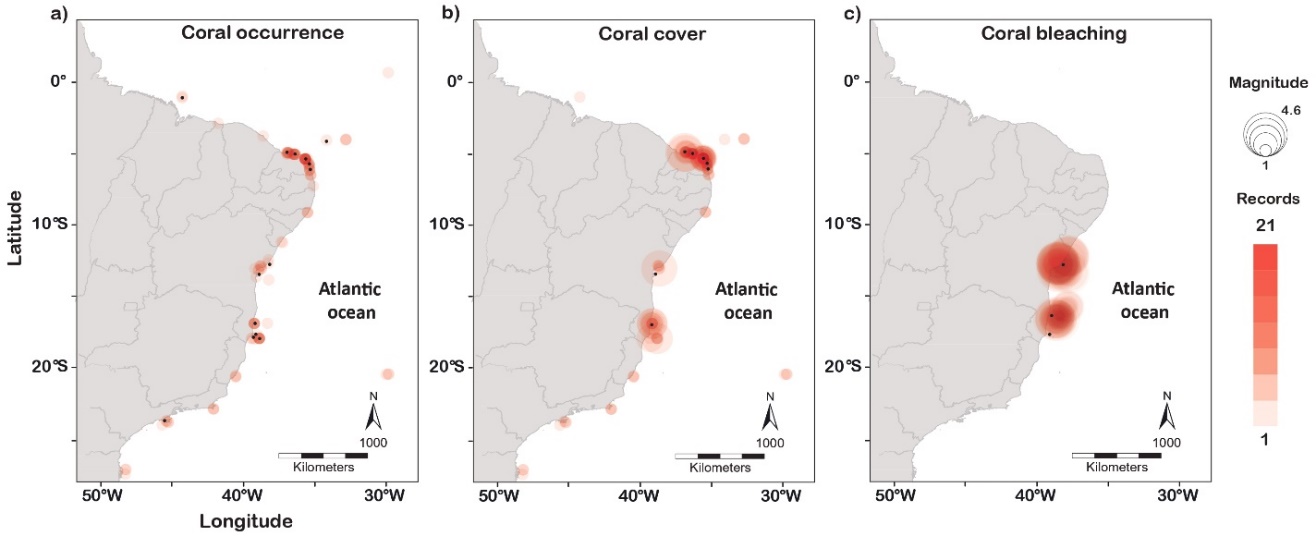


**Figure S3.** Dataset used in this study for coral occurrence (a), coral cover (b), and coral bleaching (c). The color intensity of the circles represents the number of records at each site and the size represents the magnitude of the data (coral occurrence = 1; coral cover and bleaching = log of data that ranges from 0 to 100). Sites that include data outside the temporal range of current environmental data by Bio-ORACLE (2000-2014) are shown with a black dot. Maps were plotted using the package “maptools”^3^ in R software^2^.

**Environmental variables**

For current projections we selected eight environmental variables extracted from the BIO-ORACLE database with a temporal resolution of 2000 to 2014. For future projections, we extracted Sea Surface Temperature (SST) and Sea Surface Salinity (SSS) under the RCP8.5 for two time periods, 2040-2050 and 2090-2100. The eight variables selected as predictors in the models can determine suitable habitats for coral species in which each of the variables composes a dimension of the multi-dimensional ecological niche of coral species. These conditions are essential for hermatypic corals survival, growth and reproduction, and unusual variations in these parameters tend to jeopardize coral´s performance^4^. Among the oceanographic variables: temperature (SST) and salinity (SSS) are linked to coral bleaching^5–7^, the diffused attenuation coefficient (KD) indicates the sunlight penetration in the water column^8,9^; pH, dissolved oxygen (O_2_) and net primary productivity (PP) are essential for coral growth, metabolism and food availability^10–12^. Regarding the topographic variables, bathymetry is related to light availability^13^ and the roughness of the seafloor as a proxy for rugosity indicates hard bottom structures such as reefs^14^, both essential to determine coral occurrence. All variables where standardized before included in the models.

**Modeling approach**

**Spatial effect with Delaunay triangulation**

The basic idea is to deal with the species’ occurrence, cover and bleaching (one independent model for each of these variables) at a new location as a random variable with a certain probability of success and to calculate a point estimation of this probability, and even its full predictive density. INLA uses the stochastic partial differential equation (SPDE) module for the spatial effect, which has a handful of functions to create prediction locations. For instance, it allows the construction of a Delaunay triangulation^15^ covering the region (Fig. S4). As opposed to a regular grid, a triangulation is a partition of the region into triangles, satisfying constraints on their size and shape in order to ensure smooth transitions between large and small triangles. Initially, observations are treated as initial vertices for the triangulation, and extra vertices are added heuristically to minimize the number of triangles needed to cover the region subject to the triangulation constraints. These extra vertices are used as prediction locations. Statistically, this approach has at least two advantages over a regular grid: (i) the triangulation is denser in regions where there are more observations and consequently there is more information to inform predictions; and (ii) it saves computing time, because prediction locations are typically much lower in number than those in a regular grid. Once the prediction is performed in the selected location, there are additional functions that linearly interpolate the results within each triangle into a finer regular grid. As a result of the process, a faceted surface prediction is obtained which approximates to the true predictive surface. The prediction in INLA is performed simultaneously with the inference, considering the prediction locations as points where the response is missing. The triangulation method allows predictions at much finer and more relevant biological scales, particularly where there are more data points. This allows predictions and interpolations to be based on meso-regional or local environmental data, accounting for the neighboring relationships that reduce border effect and enhance the biological relevance of relationships.


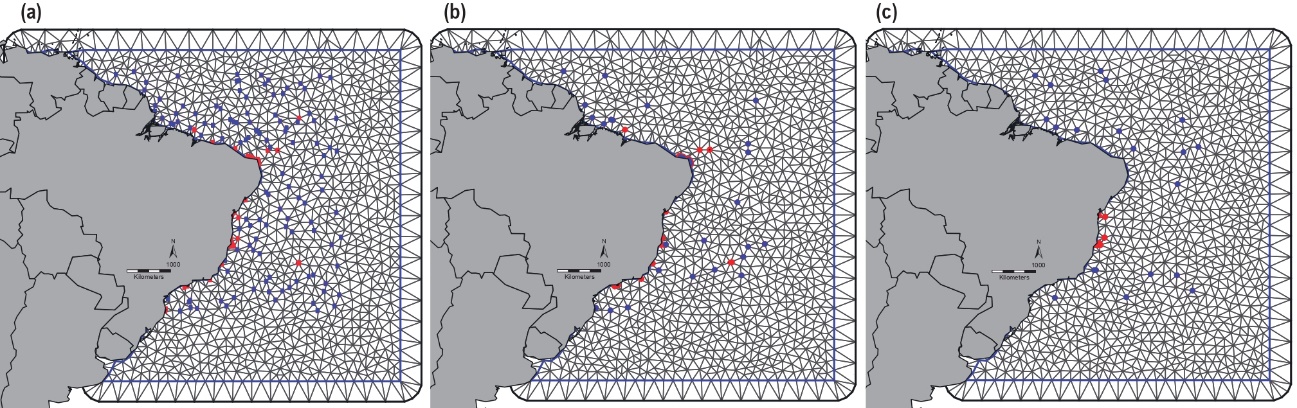


**Figure S4.** Triangulation used to calculate Gaussian Markov Random Field (GMRF) for the Stochastic Partial Differential Equations (SPDE) approach. Red dots represent presence of data of coral occurrence (a), cover (b) and bleaching (c) and blue dots represent absence of data.

**Supplementary tables**

**Table S1.** Predicted cumulative probability and percentage change between projections.

| Projection | Year of projection | Cumulative probability | % change |
| --- | --- | --- | --- |
| Coral occurrence | 2000-2014 | 0.593989 | - |
|  | 2040-2050 | 0.661154 | 11.3% |
|  | 2090-2100 | 0.687633 | 15.8% |
| Coral cover | 2000-2014 | 0.668619 | - |
|  | 2040-2050 | 0.743139 | 11.1% |
|  | 2090-2100 | 0.741318 | 10.9% |
| Coral bleaching | 2000-2014 | 0.689966 | - |
|  | 2040-2050 | 0.708667 | 2.7% |
|  | 2090-2100 | 0.705920 | 2.3% |
| Vulnerability | 2000-2014 | 0.353823 | - |
|  | 2040-2050 | 0.406858 | 15% |
|  | 2090-2100 | 0.403135 | 13.9% |

**Table S2.** List of all coral species searched to build our biological data set.

| **Coral species** |
| --- |
| *Agaricia agaricites** |
| *Agaricia fragilis** |
| *Agaricia humilis** |
| *Favia gravida* |
| *Madracis decactis* |
| *Meandrina braziliensis* |
| *Millepora alcicornis* |
| *Millepora braziliensis* |
| *Millepora laboreli* |
| *Millepora nitida* |
| *Montastraea cavernosa* |
| *Mussismilia braziliensis* |
| *Mussismilia harttii* |
| *Mussismilia hispida* |
| *Mussismilia leptophylla* |
| *Porites astreoides* |
| *Porites branneri* |
| *Scolymia cubensis** |
| *Scolymia wellsi** |
| *Siderastrea radians** |
| *Siderastrea siderea** |
| *Siderastrea stellata** |
| *Stephanocoenia michelini* |

*Species data inserted by genera in the dataset due to potential taxonomic uncertainties.

**Table S3.** Site, year and citation of articles used to build our biological data set.

| **Study area** | **Spatial distribution** | **N of sites** | **N of samples** | **Year of sample** | **Method** | **Accessed data** | **Used data** | **Ref.** |
| --- | --- | --- | --- | --- | --- | --- | --- | --- |
| Manuel Luiz Marine State Park | 00°46'S, 44°15'W | 1 | 1 sample (28 calcified hydrocorals, 66 scleractinian corals) | 1998 | Samples and observations | In text | Occurrence | ^16^ |
| Brazilian province | 0° - 27°S | 40 sites (in 15 localities) | 3820 (photoquadrats) | 2011-2014 | Photoquadrats (25x25cm) | Raw data | Occurrence and mean cover | ^17^ |
| Brazilian province | 0° - 27°S | 19 | Species list from 7 source papers | - | - | Table | Occurrence | ^18^* |
| Abrolhos bank – Viçosa Reef | 17°58.26''S – 39°15.371''W | 1 | 9 scleractinian corals species; bleaching % of 3 coral species; more than 20 specimens in 100m² | 1993-1996 | Percentage bleaching based on count of coral colonies in quadrats of 100m² | In text and tables | Occurrence and bleaching | ^19^ |
| Itacolomis Reefs | 16°53,5'S, 39°03,8'W | 1 site (6 stations) | 3 samples in each station; 11 scleractinian coral species | 1997 | Point line intersection transects (5 to 10 transects of 5m long, 175 points in each transect) | Tables | Occurrence and mean cover | ^20^ |
| Seixas Reefs | 7°09'21''S, 34°47'10''W | 1 | Bleaching record of 26 scleractinian corals (4 species) and 10 calcified hydrocorals (1 species) | 2010 | Count of colonies in 200m² | In text | Occurrence (based on bleaching records) | ^21^ |
| Todos os Santos Bay | 13°07'S, 38°44'W | 8 | 6 transects | 2003 | Video-transect (20 x 0.21m) | In text and tables | Occurrence and mean cover | ^22^ |
| Northern of Bahia state | 12°34'42''S - 12°40'04''S, 37°58'59''W - 38°04'47''W | 4 | 2380 quadrats (140 per year) | 1997-2011 | Quadrats (35lm²) | Graphs | Occurrence, mean cover and bleaching % | ^23^** |
| Todos os Santos Bay, Tinharé Island and Abrolhos Reefs | 13°07'S, 38°44'W; 13°48'S, 38°91'W; 18°00’S, 39°00'W | 3 | 3 to 6 transects; 6 video-transects | 2002-2003 | Adapted AGRRA benthos Protocol –transects (10x1m); vídeo-transect (5m²) | In text and tables | Occurrence | ^24^ |
| Santa Bárbara Island and Parcel dos Abrolhos Chapeirões |  | 8 |  | 2000 | AGRRA benthos Protocol 2.0 | Tables | Occurrence and % bleaching | ^25^ |
| Coast of Bahia state (Cabralha/Porto Seguro reefs, Abrolhos reef complex); and Brazilian Province | 13°S-39°W; 18°S-39°W; 0°S-27° | 2 | 7 samples; and 4 source papers | 1997-1998 | Surveys – bleaching categories | In text and tables | Occurrence and % bleaching | ^26^ |
| Caramuanas Reefs (BTS), Tinharé and Biopeba Islands, Itacolomis reefs, Islands of the archipelago of Abrolhos and Chapeirões of Parcel dos Abrolhos |  | 16 | 106 transects | 1998-2005 | AGRRA benthos Protocol – 5 quadrats (25x25cm) in each transect line (10m) | In text and tables | Occurrence and % bleaching | ^27^ |
| Brazilian province | 0°S-25°S | 16 | 12 source papers | 1962-2010 | Species list from 12 source papers | In text table | Occurrence | ^28^******* |
| São Sebastião Channel | 23°49'43''S,45°25'18''W | 1 |  | 1994 | Observations | In text | Occurrence | ^29^ |
| Caramuanas Reefs | 13°07'S,38°43'W; 13°07'S,38°44'W; 13°08'S,38°44'W | 1 site (3 stations) | 12 transects | 2009-2010 | Adapted from BLAGRRA protocol – transects (20x1m) | In text, tables and graphs | Occurrence and % bleaching | ^30^ |
| Rocas Atoll | 3°50'S, 33°49'W | 1 | 250 photoquadrats | 2016-2017 | Photoquadrats (60x60cm) | In text | Occurrence | ^31^ |
| Reefs of Rio Grande do Norte state | 04°44'58.42"S, 36°39'57.79"W - 06°22'44.3''S, 34°58'13.4''W | 45 | 3030 photoquadrats (30 random points at each) | 2016-2017 | Belt-transect (20x2m) - quadrats of 25x20cm | Raw data | Occurrence and mean cover | ^32^ |
| Abrolhos Reefs (Pedra Lixa, Pedra de Leste, Sebastião Gomes, Coroa Vermelha, Viçosa) | 17°26,204' S, 39°08,038' W;  17°41,535' S, 38°58,608' W;  17°47, 169' S, 39°02,979' W;  17°54, 158' S, 39°07,568' W;  17°58,004' S,  39° 11,889' W;  17°58,873'S  039°15,338' W; | 5 sites (16 stations) | 8 point intercept transect per station | 2001-2002 | Point  intercept transects (250 random points in 10m) | In text and tables | Occurrence and mean cover | ^33^ |
| Abrolhos Reefs (Pedra de Leste, Ponta Sul and Parcel dos Abrolhos) | 17°47.261’S, 39°02.795’W;  17°53.025’S,  38°59.265’W;  17°58.203’S, 38°40.230’W | 3 | 45 transects (15 per site) | 1999-2001 | Point  intercept transects | In text and tables | Occurrence and mean cover | ^34^ |

*Cape, 2012 - included data: ^26,35–40^ ; **Kelmo & Atrill, 2013- included data: ^41^ ; ***Leão et al., 2016 – included data: ^16,20,35,38–40,42–45^

**Table S4.** Predictive variables, spatial resolution, temporal range, and platforms where they were taken.

| **Predictor** | **Spatial resolution** | **Value** | **Temporal range** | **Platform** |
| --- | --- | --- | --- | --- |
| SST (°C) | 0.08 x 0.08 degrees | Mean | 2000-2014 | http://www.bio-oracle.org |
| SSS (PSU) | 0.08 x 0.08 degrees | Mean | 2000-2014 | http://www.bio-oracle.org |
| KD (m) | 0.08 x 0.08 degrees | Mean | 2000-2014 | http://www.bio-oracle.org |
| pH | 0.08 x 0.08 degrees | Mean | 2000-2014 | http://www.bio-oracle.org |
| O_2_ | 0.08 x 0.08 degrees | Mean | 2000-2014 | http://www.bio-oracle.org |
| PP | 0.08 x 0.08 degrees | Mean | 2000-2014 | http://www.bio-oracle.org |
| Bathymetry (m) | 0.01 x 0.01 degrees | Mean | 2000-2014 | <http://www.marspec.org> |
| Rugosity (m) | 0.01 x 0.01 degrees | Mean | 2000-2014 | Derived from the Bathymetry raster |
| SST (°C) | 0.08 x 0.08 degrees | Mean | 2040-2050 | http://www.bio-oracle.org |
| SSS (PSU) | 0.08 x 0.08 degrees | Mean | 2040-2050 | http://www.bio-oracle.org |
| SST (°C) | 0.08 x 0.08 degrees | Mean | 2090-2100 | http://www.bio-oracle.org |
| SST (PSU) | 0.08 x 0.08 degrees | Mean | 2090-2100 | http://www.bio-oracle.org |

Predictors acronyms are: SST, sea surface temperature; SSS, sea surface salinity; PP, net primary productivity; O2, dissolved oxygen; KD, diffuse attenuation coefficient.

**Table S5.** Environmental variables used in each model, direction of effect and the mean estimated parameter.

| **Models** | **SST** | | **SSS** | | **pH** | | **KD** | | **Bathymetry** | | **Rugosity** | |
| --- | --- | --- | --- | --- | --- | --- | --- | --- | --- | --- | --- | --- |
|  | **direction** | **MEP** | **direction** | **MEP** | **direction** | **MEP** | **direction** | **MEP** | **direction** | **MEP** | **direction** | **MEP** |
| Coral occurrence | - | 6.2 | + | 2.6 | + | 1.2 | + | 2.2 |  |  | + | 1.1 |
| Coral cover | + | 2.4 | + | 4.6 | - | 0.6 | + | 1.8 | + | 2.5 |  |  |
| Coral bleaching | + | 0.10 | + | 0.10 | - | 0.04 | + | 0.01 |  |  | + | 0.12 |

Statistic acronyms: MEP, mean estimated parameter; Predictors acronyms: Sea Surface Temperature, SST; Sea Surface Salinity, SSS; pH; Diffuse Attenuation Coefficient, KD.

**Table S6.** Comparison of the seven most relevant models for each variable based on the lowest Watanabe Akaike Information Criteria (WAIC) and Log-Conditional Predictive Ordinates (LCPO) values. Blue-shaded models in bold indicate the Best-fit (WAIC) and the best predictive quality (LCPO) model selected for each variable. W = spatial effect.
The column “Failures”, indicates the sum of the failure vector calculated by the internal leave-one-out cross validation of R-INLA.

| **Model** | | **WAIC** | **LCPO** | **Failure** |
| --- | --- | --- | --- | --- |
| Coral occurrence | **KD + pH + SSS + SST + Rugosity + W** | **882.84** | **46.50** | **0.12** |
|  | pH + SSS + SST + Rugosity + W | 2629.11 | 61.13 | 0.34 |
|  | KD + SSS + SST + Rugosity + W | 967.52 | 58.72 | 0.20 |
|  | KD + PH + SSS + SST +W | 2237.18 | 60.47 | 0.12 |
|  | SSS + SST + Rugosity + W | 1779.58 | 58.65 | 0.22 |
|  | KD + SSS + SST + W  SSS + SST + W | 1228.07  1804.68 | 62.38  58.35 | 0.14  0.23 |
| Coral cover | **Bathy + KD + pH + SSS + SST + W** | **548.20** | **2.68** | **0.08** |
|  | Bathy + KD + SSS + SST + W | 549.61 | 2.33 | 0.15 |
|  | Bathy + SSS + SST + W | 552.09 | 2.18 | 0.12 |
|  | KD + pH + SSS + SST + W | 559.69 | 5.15 | 0.18 |
|  | KD + SSS + SST + W | 550.54 | 3.55 | 0.10 |
|  | pH+ SSS + SST + W  SSS + SST + W | 577.53  566.50 | 6.91  5.87 | 0.18  0.20 |
| Coral bleaching | **KD + pH + SSS + SST + Rugosity + W** | **595.01** | **2.05** | **0.13** |
|  | pH + SSS + SST + Rugosity + W | 624.96 | 3.20 | 0.15 |
|  | KD + SSS + SST + Rugosity + W | 603.37 | 3.25 | 0.16 |
|  | KD + PH + SSS + SST +W | 737.39 | 4.53 | 0.14 |
|  | SSS + SST + Rugosity + W  KD + SSS + SST + W | 720.90  780.15 | 4.67  5.76 | 0.26  0.24 |
|  | SSS + SST + W | 823.42 | 5.03 | 0.20 |

**Table S7.** Numerical summary of the posterior distributions of the fixed effects for the best model of the coral occurrence, coral cover, and coral bleaching. This summary contains the mean, the standard deviation (sd), the median (Q50) and a 95% credible interval (Q2.5 - Q97.5), which is a central interval containing 95% of the probability under the posterior distribution.

| Model | Predictor | Mean | s.d. | Q2.5 | Q50 | Q97.5 |
| --- | --- | --- | --- | --- | --- | --- |
| Coral occurrence | b0 | -3.341 | 23.194 | -50.067 | -4.079 | 46.283 |
|  | KD | 2.272 | 3.819 | -5.660 | 2.160 | 10.622 |
|  | pH | 1.213 | 5.806 | -10.737 | 0.969 | 14.062 |
|  | SSS | 2.665 | 11.368 | -21.876 | 2.795 | 26.645 |
|  | SST | -6.281 | 8.810 | -25.795 | -5.730 | 11.282 |
|  | Rugosity | 1.564 | 1.452 | -1.710 | 1.066 | 4.412 |
| Coral cover | b0.bin  b0.con | -3.808  -5.516 | 2.040  8.345 | -8.038  -21.993 | -3.760  -5.499 | 0.157  10.851 |
|  | Bathy.bin  Bathy.con | 2.242  2.526 | 0.931  3.440 | 0.490  -4.226 | 2.200  2.520 | 4.221  9.307 |
|  | KD.bin  KD.con | 1.306  1.857 | 0.661  0.910 | 0.048  0.075 | 1.291  1.853 | 2.652  3.658 |
|  | pH.bin  pH.con | 1.445  -0.680 | 1.280  2.396 | -0.994  -5.375 | 1.389  -0.690 | 4.196  4.067 |
|  | SSS.bin  SSS.con | 2.205  4.683 | 1.398  2.674 | -0.509  -0.520 | 2.173  4.658 | 5.088  10.032 |
|  | SST.bin  SST.con | 0.446  2.465 | 0.861  1.409 | -1.251  -0.213 | 0.409  2.426 | 2.323  5.378 |
| Coral bleaching | b0 | -0.119 | 0.140 | -0.425 | -0.118 | 0.195 |
|  | KD | 0.016 | 0.022 | -0.032 | 0.017 | 0.056 |
|  | pH | -0.043 | 0.050 | -0.141 | -0.044 | 0.057 |
|  | SSS | 0.104 | 0.071 | -0.044 | 0.107 | 0.236 |
|  | SST | 0.100 | 0.080 | -0.060 | 0.100 | 0.265 |
|  | Rugosity | 0.125 | 0.095 | -0.063 | 0.124 | 0.314 |

Acronyms: .bin for binary and .con for continuous

**Table S8.** Summary of the probability of each estimated beta to be different from 0 computed with the posterior marginal distributions.

| Model | Predictor | P(beta ≠0) |
| --- | --- | --- |
|  | KD | 0.929794 |
|  | pH | 0.724038 |
| Coral occurrence | SSS | 0.819008 |
|  | SST | 0.734078 |
|  | Rugosity | 0.892292 |
| Coral Cover | Bathy.bin  Bathy.con | 1  0.838941 |
|  | KD.bin  KD.con | 0.992982  0.985154 |
|  | pH.bin  pH.con | 0.968677  0.583898 |
|  | SSS.bin  SSS.con | 0.999634  0.977057 |
|  | SST.bin  SST.con | 0.535939  0.972290 |
| Coral bleaching | KD | 0.6362391 |
|  | pH | 0.6398997 |
|  | SSS | 0.8166243 |
|  | SST | 0.6265565 |
|  | Rugosity | 0.7070776 |

Acronyms: .bin for binary and .con for continuous

**References**

1. Phillips, N. A Companion to the e-Book ``YaRrr!: The Pirate’s Guide to R’’. (2017).

2. R Core Team. R: A Language and Environment for Statistical Computing. (2020).

3. Bivand, R. & Nicholas, L.-K. Package ‘maptools’: Tools for Handling Spatial Objects. (2020).

4. Kleypas, J. A., McManu, J. W. & Mene, L. A. B. Environmental limits to coral reef development: Where do we draw the line? *Am. Zool.* **39**, 146–159 (1999).

5. Hoegh-Guldberg, O. The future of coral reefs : integrating climate model projections and the recent behaviour of corals and their dinoflagellates. *Proc. 9th Int. Coral Reef Symp.* 1–6 (2000).

6. Hoegh-Guldberg, O. & Fine, M. Low temperatures cause coral bleaching. *Coral Reefs* **23**, 444 (2004).

7. Coles, S. L. & Jokiel, P. L. Effects of salinity on coral reefs. 147–166 (1992).

8. Mies, M. *et al.* South Atlantic Coral Reefs Are Major Global Warming Refugia and Less Susceptible to Bleaching. *Front. Mar. Sci.* **7**, 1–13 (2020).

9. Sully, S. & van Woesik, R. Turbid reefs moderate coral bleaching under climate-related temperature stress. *Glob. Chang. Biol.* **26**, 1367–1373 (2020).

10. Shashar, N., Cohen, Y. & Loya, Y. Extreme Die1 Fluctuations of Oxygen in Diffusive Boundary Layers Surrounding Stony Corals. *Biol. Bull.* **185**, 455–461 (1993).

11. Hoegh-Guldberg, O. *et al.* Coral reefs under rapid climate change and ocean acidification. *Science* **318**, 1737–1742 (2007).

12. Fox, M. D., Elliott Smith, E. A., Smith, J. E. & Newsome, S. D. Trophic plasticity in a common reef-building coral: Insights from δ13C analysis of essential amino acids. *Funct. Ecol.* **33**, 2203–2214 (2019).

13. Gattuso, J. P. *et al.* Light availability in the coastal ocean: Impact on the distribution of benthic photosynthetic organisms and their contribution to primary production. *Biogeosciences* **3**, 489–513 (2006).

14. Dunn, D. C. & Halpin, P. N. Rugosity-based regional modeling of hard-bottom habitat. *Mar. Ecol. Prog. Ser.* **377**, 1–11 (2009).

15. Hjelle, Ø. & Dæhle, M. Algorithms for Delaunay Triangulation. in *Triangulations and Applications* (Springer Berlin Heidelberg, 2006).

16. Amaral, F. D., Hudson, M. & Steiner, A. Note on the Widespread Bleaching Observed At the Manuel Luiz Marine State Park, Maranhão, Brazil. *Arq. Ciências do Mar* **39**, 138–141 (2006).

17. Aued, A. W. *et al.* Large-scale patterns of benthic marine communities in the brazilian province. *PLoS One* **13**, 1–15 (2018).

18. Capel, K. C. C. Scleractinia (Cnidaria: anthozoa) da reserva biológica marinha do Arvoredo (SC), com ênfase na estrutura espaço-temporal da formação mais meridional de corais recifais no oceano Atlântico. *Экономика Региона* (2012).

19. Castro, C. B. & Pires, D. O. A bleaching event on a Brazilian coral reef. *Brazilian J. Oceanogr.* **47**, 87–90 (1999).

20. Castro, C. B., Amorim, L. C. de, Calderon, E. N. & Segal, B. Cobertura e recrutamento de corais recifais (Cnidaria: Scleractinia e Milleporidae) nos recifes Itacolomis, Brasil. *Arq. do Mus. Nac.* **64**, 29–40 (2006).

21. Dias, T. L. P. & Gondim, A. I. Bleaching in scleractinians, hydrocorals, and octocorals during thermal stress in a northeastern Brazilian reef. *Mar. Biodivers.* (2016). doi:10.1007/s12526-015-0342-8

22. Dutra, L. X. C., Kikuchi, R. K. P. & Leão, Z. M. a. N. Todos os Santos Bay coral reefs , Eastern Brazil , revisited after 40 years. *Proc. 10th Int. Coral Reef Symp.* **1095**, 1090–1095 (2006).

23. Kelmo, F. & Attrill, M. J. Severe Impact and Subsequent Recovery of a Coral Assemblage following the 1997-8 El Niño Event: A 17-Year Study from Bahia, Brazil. *PLoS One* **8**, (2013).

24. Kikuchi, R. K. P. *et al.* Branqueamento de corais nos recifes da Bahia associado aos efeitos do El Niño 2003 Laboratório de Estudos Costeiros , Centro de Pesquisas em Geofísica e Geologia , Universidade Federal da Resumo : Este trabalho apresenta os resultados preliminares do acom. *Coral Reefs* 2–4 (2003).

25. Kikuchi, R. K. P., Leao, Z. M. A. N., Testa, V., Dutra, L. X. C. & Spano, S. Rapid Assessment of the Abrolhos Reefs, Eastern Brazil (Part 1: Stony Corals and Algae). *Atoll Res. Bull.* **496**, 172–187 (2003).

26. Leão, Z. M. A. N., Kikuchi, R. K. P. & Testa, V. *Corals and coral reefs of Brazil*. *Latin American Coral Reefs* (2003). doi:10.1016/B978-044451388-5/50003-5

27. Leão, Z. M. A. N., De Kikuchi, R. K. P. & De Oliveira, M. D. D. M. Coral bleaching in Bahia reefs and its relation with sea surface temperatura anomalies. *Biota Neotrop.* **8**, 69–82 (2008).

28. Leão, Z. M. A. N. *et al.* Brazilian coral reefs in a period of global change: A synthesis. *Brazilian J. Oceanogr.* **64**, 97–116 (2016).

29. Migotto, A. E. Anthozoan bleaching on the southeastern coast of Brazil in the summer of 1994. *Int. Conf. Coelenterate Biol.* 329–335 (1997).

30. Miranda, R. J., Cruz, I. C. S. & Leão, Z. M. A. N. Coral bleaching in the Caramuanas reef (Todos os Santos Bay, Brazil) during the 2010 El Niño event. *Lat. Am. J. Aquat. Res.* **41**, 351–360 (2013).

31. Ozekoski, R. Branqueamento e recrutamento de Siderastrea stellata no Atol das Rocas - Brasil, durante e após o ENSO 2015-2016. 44 (2017).

32. Roos, N. C., Pennino, M. G., Carvalho, A. R. & Longo, G. O. Drivers of abundance and biomass of Brazilian parrotfishes. *Mar. Ecol. Prog. Ser.* **623**, 117–130 (2019).

33. Segal-Ramos, B. Corais e comunidades recifais e sua relação com a sedimentação no Banco de Abrolhos, Brasil. *Tese* 133 (2003).

34. Segal, B. & Castro, C. B. Coral community structure and sedimentation at different distances from the coast of the Abrolhos Bank, Brazil. *Brazilian J. Oceanogr.* **59**, 119–129 (2011).

35. Amaral, F. D., Hudson, M. M., Steiner, A. Q. & Ramos, C. A. C. Corals and calcified hydroids of the Manuel Luiz Marine State Park (State of Maranhão, Northeast Brazil). *Biota Neotrop.* **7**, 73–81 (2007).

36. Castro, C. B.; Pires, D. BRAZILIAN CORAL REEFS: WHAT WE ALREADY KNOW AND WHAT IS STILL MISSING Clovis B. Castro and Débora O. Pires. *Bull. Mar. Sci.* **69**, 357–371 (2001).

37. Couto, E. C. G., Silveira, F. L. Da & Rocha, G. R. a. Marine biodiversity in Brazil: the currents status. *Gayana* **67**, 327–340 (2003).

38. Neves, E., Johnsson, R., Sampaio, C. & Pichon, M. The occurrence of Scolymia cubensis in Brazil: revising the problem of the Caribbean solitary mussids. *Zootaxa* **1366**, 45–54 (2006).

39. Neves, E. G., Andrade, S. C. S., Da Silveira, F. L. & Solferini, V. N. Genetic variation and population structuring in two brooding coral species (Siderastrea stellata and Siderastrea radians) from Brazil. *Genetica* **132**, 243–254 (2008).

40. Neves, E. G., da Silveira, F. L., Pichon, M. & Johnsson, R. Cnidaria, Scleractinia, Siderastreidae, Siderastrea siderea (Ellis and Solander, 1786): Hartt Expedition and the first record of a Caribbean siderastreid in tropical Southwestern Atlantic. *Check List* **6**, 505–510 (2010).

41. Kelmo, F., Attrill, M. J. & Jones, M. B. Effects of the 1997-1998 El Niño on the cnidarian community of a high turbidity coral reef system (northern Bahia, Brazil). *Coral Reefs* **22**, 541–550 (2003).

42. Amaral, F. M. D., Steiner, A. Q., Broadhurst, M. K. & Cairns, S. D. An overview of the shallow-water calcified hydroids from Brazil (Hydrozoa: Cnidaria), including the description of a new species. *Zootaxa* **68**, 56–68 (2008).

43. Amaral, F. M. D. *et al.* Checklist and morphometry of benthic cnidarians from the Fernando de Noronha Archipelago, Brazil. *Cah. Biol. Mar.* **50**, 277–290 (2009).

44. Oigman-Pszczol, S. S. & Creed, J. C. Size structure and spatial distribution of the corals Mussismilia hispida and Siderastrea stellata (Scleractinia) at Armação dos Búzios, Brazil. *Bull. Mar. Sci.* **74**, 433–448 (2004).

45. Oigman-Pszczol, S. S. & Creed, J. C. Distribution and abundance of fauna on living tissues of two Brazilian hermatypic corals (Mussismilia hispida (Verril 1902) and Siderastrea stellata Verril, 1868). *Hydrobiologia* **563**, 143–154 (2006).
